# Creep Failure Characteristics and Mathematical Modeling of High-Density Polyethylene Geomembranes under High Stress Levels

**DOI:** 10.3390/polym16142019

**Published:** 2024-07-15

**Authors:** Libo Wang, Weijun Cen, Erich Bauer, Jiangliang Wei, Zhenyu Wen, Jun Yan

**Affiliations:** 1 College of Water Conservancy and Hydropower Engineering, Hohai University, Nanjing 210098, China; wlb666@hhu.edu.cn (L.W.); 221302020028@hhu.edu.cn (J.W.); wen_zhenyu@ctg.com.cn (Z.W.); 2 Institute of Applied Mechanics, Graz University of Technology, 8010 Graz, Austria; erich.bauer@tugraz.at; 3 Department of Geotechnical Engineering, China Institute of Water Resources and Hydropower Research, Beijing 100083, China; yanjun@iwhr.com

**Keywords:** geosynthetics, HDPE geomembranes, high stress level, creep characteristics, creep rupture, mathematical modeling

## Abstract

To explore the creep characteristics of geomembrane under different tensile stresses, a series of creep tests were carried out on high-density polyethylene (HDPE) geomembrane specimens. For the interpretation and fitting of the experimental data, refined approximation functions were proposed. Particular attention was paid to the creep failure behavior under high tensile stresses, i.e., 70%, 80%, and 90% of maximum peak stress. To investigate the effects of size on the mechanical response, experiments with two different membrane thicknesses were conducted. The results obtained under high stress levels were compared with creep tests at medium and low stress levels. Depending on load level, different creep characteristics can be distinguished. In the secondary creep state, the creep velocity is higher for higher load levels. In contrast to the medium and low load levels, the geomembrane under high stresses underwent the tertiary creep stage after instantaneous deformation and primary and secondary creep stages. In some tests, it was observed that under very high stress levels, creep velocity does not necessarily follow the expected trend and creep rupture can occur within a short time. For numerical simulation, an improved mathematical model was proposed to reproduce in a unified manner the experimental data of the whole non-linear evolution of creep elongation under different stress levels.

## 1. Introduction

High-density polyethylene (HDPE) geomembranes are widely used in many hydraulic and environmental geotechnical applications like, for instance, water reservoirs, leaching ponds, landfills, tunnels, and water canal construction [1,2,3,4]. As barriers against liquid and gas flow, geomembranes are often buried between cushion and protective layers composed of sand or soil in many applications. The material exhibits creep behavior when subjected to long-term loads, such as water pressure, gravitational force of municipal solid waste, and interfacial friction. Large tensile deformation of geomembranes also tends to occur at local contact areas of concave or convex neighboring underlayers and anchoring expansion joints.

In particular, under high stress levels, creep behavior can lead to the recombination of the intrinsic stress state and the ultimate rupturing of geomembranes [5]. As a consequence of creep rupture, water or waste liquid can pass through the damaged membrane sealing, causing a significant safety risk of anti-seepage control of geotechnical structures. Although the average maximum design stress for geomembrane structures is much lower than breaking strength, unexpected local conditions can lead to higher stress concentration and, consequently, to a reduction of the lifetime of the geomembrane [6]. Thus, investigating the long-term behavior of geomembranes under higher load levels is of practical importance with regard to the safety of geotechnical construction and the intended operation period.

**Figure 1:**
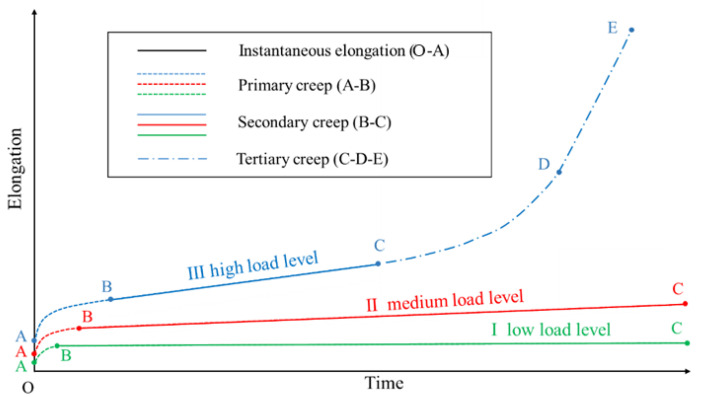
Schematic plot of typical time-dependent elongation under (I) low, (II) medium, and (III) high load levels.

The raw material of HDPE geomembrane is a high-molecular, semi-crystalline polymer formed by crystalline and amorphous regions [7,8,9,10,11]. The special structure of crystalline and interlayer amorphous chains gives HDPE geomembranes a viscoelastic-plastic material behavior [12]. Under different load levels, the creep stages of polymeric materials display various characteristics. According to Koerner et al. [13], the creep behavior of HDPE geomembranes can be divided into four stages, namely instantaneous (O–A), primary (A–B), secondary (B–C), and tertiary creep stage (C–D–E), as illustrated in Figure 1. After instantaneous elongation, polymeric materials enter the primary creep stage first. Under low load levels, creep strain tends to stagnate in the secondary creep stage, while under medium load levels, creep strain increases almost linearly with time. Under high load levels, the material enters the tertiary creep stage after maintaining a relatively constant creep rate for a period of time in the secondary stage. Creep strain in the tertiary creep stage increases sharply with time until creep rupture occurs.

Considerable research has been conducted to appraise the result of creep tests of polymer materials and to propose mathematical models to simulate the experimental data [14,15,16]. However, only a few studies focus on creep failure characteristics under high load levels [17].

From Figure 1, it is obvious that for their numerical simulation, the different creep characteristics require appropriate approximation functions. In a simplified manner, elastic spring elements and damper elements are frequently combined to simulate the one-dimensional viscoelastic material behavior [18,19]. Depending on the arrangement of spring and damper elements, particular creep characteristics can be described. However, a more detailed inspection shows that the material parameters involved also depend on the load level within the range of a considered load characteristic [20]. The present paper proposes a refined concept to describe creep behavior for the whole range of loads within a particular load characteristic using a single set of material parameters. Moreover, the size effects observed in the creep experiments are also captured by the improved model.

The paper is organized as follows: Section 2 deals with the experimental investigations of the elongation of HDPE sheets with two different thicknesses under plane stress conditions. In particular, the stress–strain behavior and peak strength of HDPE sheets with an initial size of 100 mm × 50 mm and sheet thicknesses of 0.5 mm and 1.5 mm carried out in strain-controlled tensile tests are shown in Section 2.1. The creep test equipment and the preparation of geomembrane specimens are described in Section 2.2. The results of the creep tests under high load levels of 70%, 80%, and 90% are outlined in Section 2.3. Particular attention was paid to the different stages of the non-linear evolution of creep strain. The relationship between critical creep time, failure time, and load level is under in-depth discussion. The experimental results obtained under high stress levels are compared with the results from low and medium load levels in Section 2.4. In Section 3, improved approximation functions are proposed to model creep behavior in a unified manner up to breaking elongation. Conclusions are given in Section 4.

## 2. Laboratory Investigations

### 2.1. Tensile Tests

**Figure 2:**
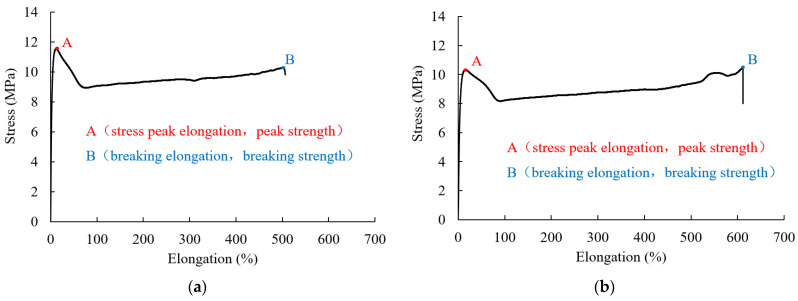
Displacement-controlled tensile test with HDPE geomembrane thicknesses of (**a**) 0.5 mm and (**b**) 1.5 mm.

In order to explore the one-dimensional tensile behavior of the HDPE geomembrane used, material displacement-controlled tensile tests with a UTM4503 tensile tester were carried out on specimens with an initial length of 100 mm, a width of 50 mm, and two different thicknesses. The HDPE geomembrane material used in the laboratory experiments was manufactured by Material Co., Ltd. in Dezhou, Shandong, China. The tests were conducted with a constant displacement velocity of 0.334 mm/s. For both specimen thicknesses, namely 0.5 mm and 1.5 mm, the stress-elongation relations are shown in Figure 2, and the quantities of peak strength, breaking strength, and corresponding elongations are summarized in Table 1. For the representation of the test results, nominal stresses and strains are considered.

It is obvious that after the stress peak, the material exhibits strain softening and subsequently strain hardening up to breaking strength. Breaking strength is only a little lower than peak strength. There is a certain difference in peak strength and breaking strength for the specimen thicknesses of 0.5 mm and 1.5 mm. The results of repeated tensile tests showed similar differences lying within a range of less than 10%. For the thicker specimen, peak elongation is a factor of 1.36, and breaking elongation is a factor of 1.21 larger than for the thinner specimen. This indicates that the thicker membrane behaves slightly more leniently under stretching. These differences may be explained by the fact that the quality of the manufactured geomembrane material is not perfectly even, and in the softening regime, the behavior is strongly influenced by inhomogeneous deformation and local plastifications. It is worth noting that for rate-dependent material, the stress-strain relation is also influenced by the prescribed loading velocity. Moreover, under higher loads, the phenomenon of significant necking and development of crazing areas locally leads to higher stresses. Thus, the value of such local stress concentrations can be much higher than the computed nominal stress shown in Figure 2.

### 2.2. Creep Test Equipment and Preparation of Geomembrane Specimens

**Figure 3:**
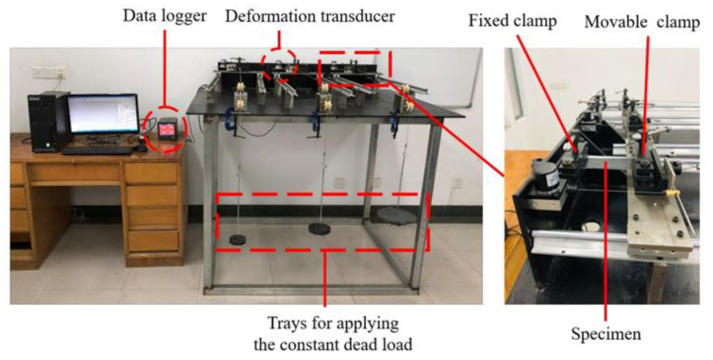
Creep test apparatus.

For creep tests on HDPE smooth geomembrane specimens with an initial length of 100 mm and an initial width of 50 mm, a dedicated test apparatus was developed at Hohai University, as shown in Figure 3. It is equipped with three different devices: the loading device, the clamping device, and the deformation measurement device. The geomembrane specimen is installed horizontally by a fixed clamp and a movable clamp. In order to avoid the influence of gravity, the clamps are mounted horizontally. The movable clamp can slide freely on the horizontal rail. The loading plate is connected to the movable clamp by a steel cable which is guided over a pulley. In each test, the weight was placed on the trays in one step and kept constant during the whole geomembrane creep test. The data acquisition device is composed of a WFS displacement sensor with a resolution of 0.1 mm and an acquisition device. The WFS displacement sensor is a product of Suzhou Fangyi Electric Co., Ltd., Suzhou, China. The acquisition device records measurements at intervals of 1 s. A personal computer is used to store the test data.

### 2.3. Creep Tests under Three Different High Stress Levels

To investigate the whole evolution of creep behavior under high stress levels, tests were conducted under constant load levels of 70%, 80%, and 90% of peak strength. In particular, the load level is defined as the ratio of the creep stress to the peak stress. The reference peak strength was taken from the tensile tests outlined in the previous subsection.

**Figure 4:**
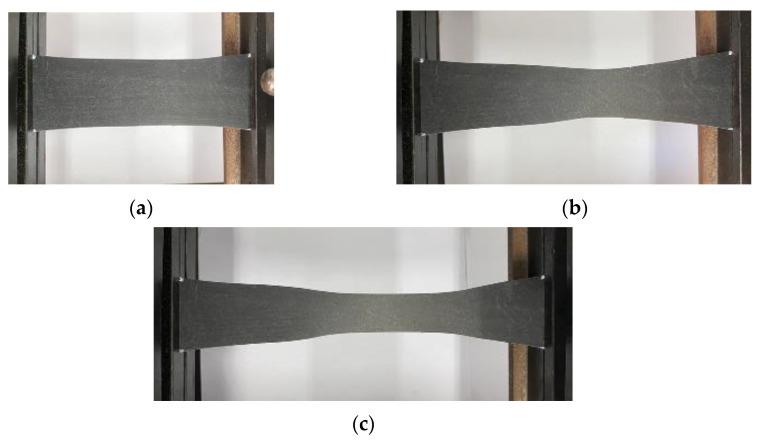
Necking phenomenon of the initially 0.5 mm thick specimen under 90% of peak load and for creep strains of approximately (**a**) 30%, (**b**) 90%, and (**c**) 150%.

The creep phenomenon of geomembrane under the three different high load levels is similar for both specimen thicknesses, 0.5 mm and 1.5 mm. A visual inspection shows that the deformation of the geomembrane is inhomogeneous from the beginning of loading caused by the rigid fixation of the end of the specimen within the clamps. In particular, in a zone in the middle of the specimen, the width becomes smaller with continued creep elongation. Figure 4 shows that for an applied load level of 90% of peak load, the phenomenon of necking already becomes dominant when specimen elongation exceeds about 30%.

**Figure 5:**
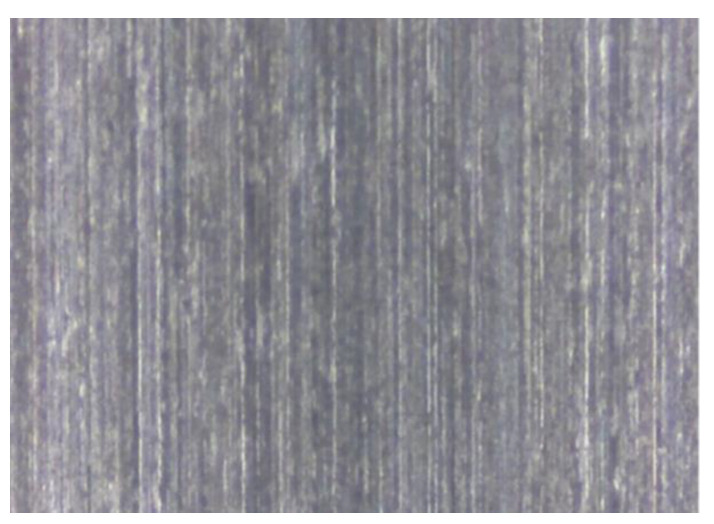
Whitening areas on a detail of the geomembrane surface before creep rupture occurs.

The appearance of whitening areas is an inherent phenomenon before the creep rupture of high polymer material, as reported by several authors [21,22,23,24,25]. In this experiment, it was observed that the whitening area on the surface of the geomembrane (Figure 5) was distributed with different sizes of crazing areas along the length of the specimen. In this context, crazing areas denote whitening areas with local micro-crack initiations. When geomembrane creep strain exceeded about 90%, the crazing area continued to fracture, manifested macroscopically as pronounced cracks. Cracks starting near internal micro voids continued to be stretched and evolved into new crazing areas. The process occurred repeatedly, and ultimately, the cracks were interconnected, resulting in macroscopic creep rupture.

**Figure 6:**
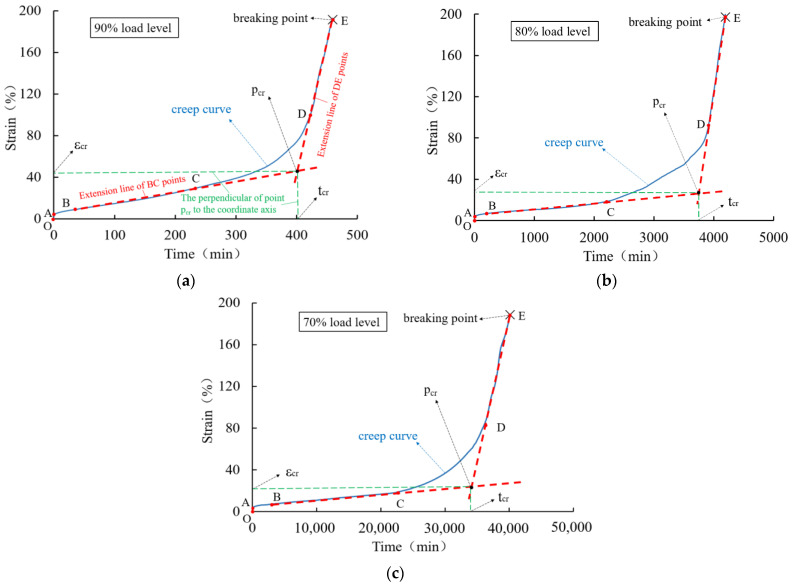
Creep curve of a 0.5 mm thick HDPE geomembrane under high load levels of (**a**) 90%, (**b**) 80%, and (**c**) 70%.

**Figure 7:**
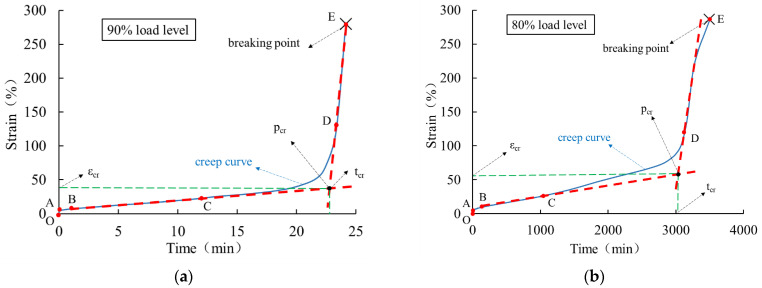

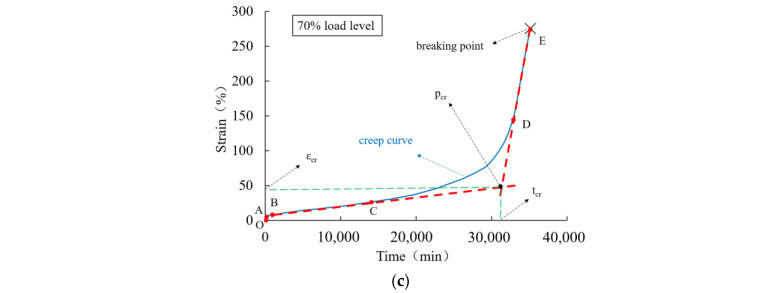
Creep curve of a 1.5 mm thick HDPE geomembrane under high load levels of (**a**) 90%, (**b**) 80%, and (**c**) 70%.

The creep behavior of HDPE geomembrane specimens under three different high load levels is shown in Figure 6 for a specimen thickness of 0.5 mm and in Figure 7 for a specimen thickness of 1.5 mm. For all three investigated load levels, the creep characteristic is qualitatively similar, but the duration until creep rupture takes place is different. Each creep curve has gone through four characteristic stages, namely instantaneous deformation (section O–A), primary creep stage (section A–B), secondary creep stage (section B–C), and tertiary creep stage (section C–E). The tertiary creep stage includes the creep transition stage (section C–D) and the rapid creep growth stage (section D–E).

**Figure 8:**
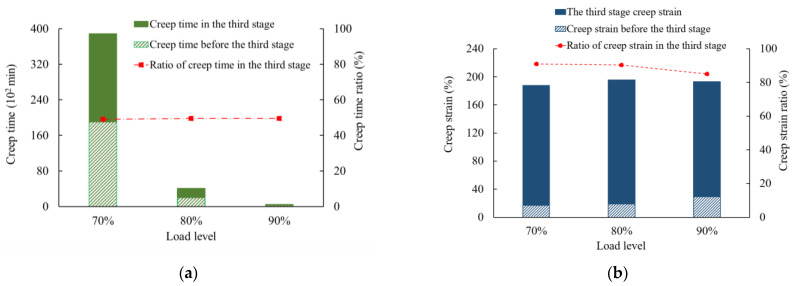

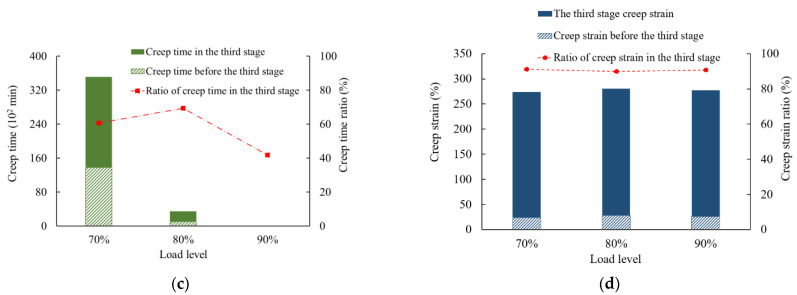
Creep time and creep strain depending on the applied load level for the initial membrane thickness of (**a**,**b**) 0.5 mm and (**c**,**d**) 1.5 mm.

Figure 8 shows the creep time and creep strain before the tertiary creep stage and in the tertiary creep stage. It is obvious that for higher load levels, creep rupture occurs significantly earlier. The creep strain at creep failure only shows moderate fluctuation but differs for different membrane thicknesses. In the third stage, namely the tertiary creep stage, creep time gradually decreases with an increase in load level. The dash-dotted lines and dotted lines denote the creep time ratio and the creep strain ratio, respectively. In particular, the creep time ratio is defined as the ratio of creep time to the time at the breaking state, and the creep strain ratio is defined as the ratio of creep strain to strain at the breaking state. Under 70%, 80%, and 90% load levels, the creep strain ratios of a 0.5 mm thick geomembrane in the tertiary stage are 90.9%, 90.3%, and 84.9%, and the creep time ratios are 49.0%, 49.5%, and 49.6%, respectively. For a 1.5 mm thick geomembrane, the creep strain ratios in the tertiary stage are 91.2%, 90.0%, and 92.0%, and the creep time ratios are 60.7%, 69.4%, and 41.9%, respectively. Therefore, the tertiary creep stage under high load levels occupies a dominant part of the whole creep process.

From Figure 6 and Figure 7, it is clear that immediately after instantaneous deformation (section O–A), time-dependent creep strain develops in a non-linear manner until creep rupture takes place. In the primary creep stage (section A–B), the curve is concave, and at the turning point B, it assumes a convex shape, indicating an increase in creep velocity. In particular, within the secondary creep stage (section B–C), creep velocity is almost constant and can be approximated in a simplified manner by the red dotted line shown in Figure 6 and Figure 7. The course of the rapid creep growth stage (section D-E) can also be approximated by a straight line. It is obvious that for all load levels, the inclination of the second line (D–E) is much steeper than that of the secondary creep stage (section B–C). Following the concept by Liu [26] and other scholars, the intersection of the extended lines is defined as the “critical creep point” (*p_cr_*), and the corresponding time (*t_cr_*) and strain (*ε_cr_*) denote the “critical creep time” and “critical creep strain”, respectively. The corresponding values under different load levels and for the 0.5 mm and 1.5 mm thick membranes are summarized in Table 2.

For a higher load level, the critical strain is higher and the critical time is lower. The values are different for different specimen thicknesses, which indicates a certain size effect of deformation behavior under high load levels. With the exception of the experimental data obtained for the 1.5 mm thick specimen under the load level of 90%, the values of creep time shorten approximately by a factor of 10 for every 10% rise in load level. Under different high load levels, the time (*t_f_*) when the creep strain of the geomembrane reaches creep failure is approximately 1.07~1.13 times the critical creep time (*t_cr_*). It is obvious that the characteristic values obtained for the particular specimen thickness of 1.5 mm under the load level of 90% are far from the trend of the other experimental results. Such behavior can be explained by local inhomogeneities leading to the instable evolution of microstructure effects, which are typical for materials with strain softening. As shown in Figure 2, strain softening is relevant for the HDPE material used, and thus, it can be concluded that for a very high load level, the stress–strain relation is no longer unique.

In Figure 6 and Figure 7, the bilinear approximation of the creep curves allows a simplified distinction between undercritical and overcritical creep behavior for practical purposes. More precisely, undercritical creep behavior is approximated by the connecting line of stages (B–C) and overcritical creep behavior by the connection between stages (D–E). The inclination of the lines estimates a measure of creep velocity in these two stages, as outlined in Table 3. Compared with the undercritical creep velocity, the overcritical creep velocity is much higher, especially at higher load levels. The values for the 1.5 mm thick specimen under the load level of 90% are out of the expected range as a result of the inhomogeneous evolution of the microstructure, as previously discussed.

From Table 2, it can be concluded that critical creep time, *t_cr_*, shows a significant downward trend with an increase in load level. The data can be fitted using the following power function:

(1) $$\sigma_{p}={at}_{cr}^{b}$$

**Figure 9:**
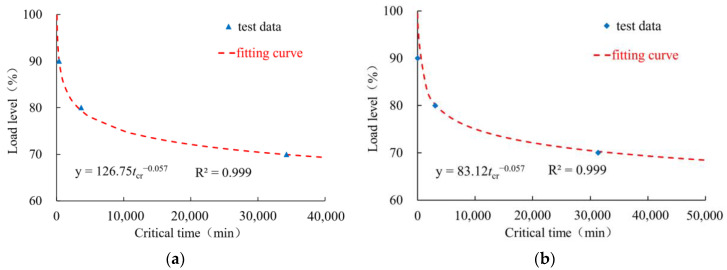
Relation between critical creep time and load level for the geomembrane thickness of (**a**) 0.5 mm and (**b**) 1.5 mm.

Here, *σ_p_* is the load level, and *a* and *b* are the fitting parameters. In particular, for the initial geomembrane thicknesses of 0.5 mm and 1.5 mm, the fitted curves are shown in Figure 9a and Figure 9b, respectively. For the different geomembrane thicknesses, the values of parameter *a* are different, which again indicates a size effect, as discussed above.

### 2.4. Comparison of Creep Curves under Low, Medium, and High Load Levels

**Figure 10:**
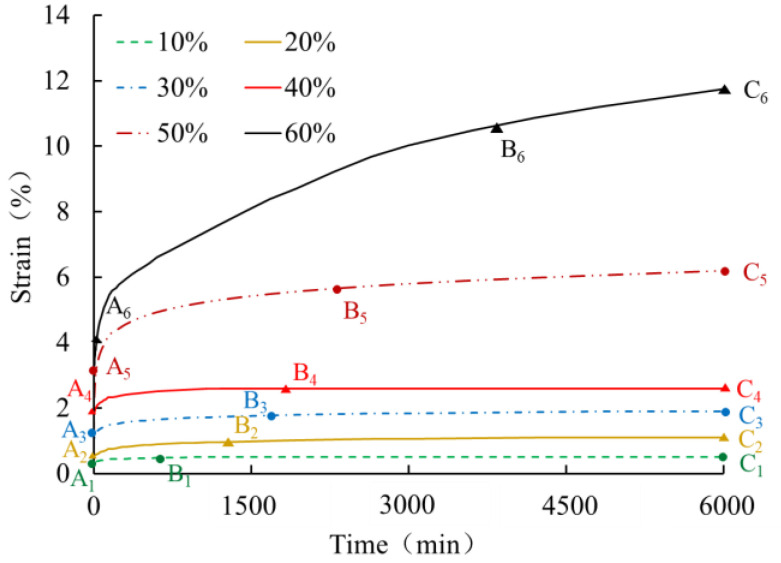
Creep curves of a 0.5 mm thick HDPE geomembrane at low and medium load levels.

The creep curves for 0.5 mm thick HDPE geomembranes obtained under low and medium load levels of 10%, 20%, 30%, 40%, 50%, and 60% are shown in Figure 10. Each of the creep curves only experiences instantaneous deformation, the primary creep stage, and the secondary creep stage, but not the tertiary creep stage. All curves exhibit a concave shape with decreasing creep velocity over time. It can, therefore, be concluded that under ordinary temperature, the creep characteristics of geomembrane typical in tertiary creep stages can only be observed under very high load levels. The experimental results for a 0.5 mm thick HDPE geomembrane reveal that under low load levels of up to 40%, creep strain after 100 h is already less than 3%, and it can thus be expected that the geomembrane will not enter the tertiary stage in a finite period of time and creep rupture will not occur within the usual lifetime of geotechnical structures.

**Figure 11:**
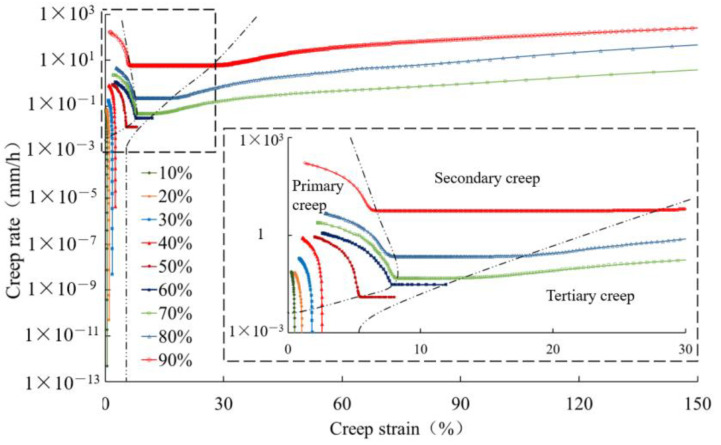
Creep rate changes for a 0.5 mm thick HDPE geomembrane under different load levels.

The geomembrane under medium load levels, i.e., between 50% and 60%, displays an almost constant rate in the secondary creep stage. In order to analyze the evolution of creep rate under low, medium, and high load levels, it is convenient to construct a Sherby–Dorn plot, as shown in Figure 11, for the 0.5 mm thick geomembrane. Independent of load level, the creep rate significantly decreases in the primary creep stage. In the secondary creep stage, the decrease in creep rate tends almost to zero under low load levels. Under high load levels, the creep rate in the secondary stage increases slightly, and in the tertiary creep stage, it increases until creep rupture takes place.

**Figure 12:**
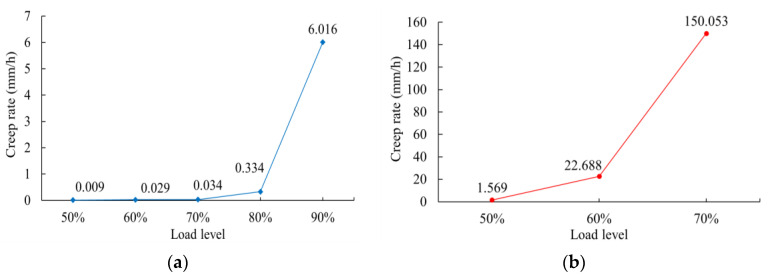
Creep rate values for a 0.5 mm thick geomembrane under different load levels: (**a**) secondary creep stage; (**b**) tertiary creep stage.

Under different load levels, a comparison of creep rates in the secondary creep stage and the rapid creep growth stage is shown in Figure 12a and Figure 12b, respectively. The creep rate in the secondary creep stage increases slowly when the load level is less than 70%, but it increases rapidly when the load level is higher than 70%. Under high load levels of 70%, 80%, and 90%, the creep rate in the secondary creep stage increases with increasing load levels. The creep rate in the rapid creep growth stage increases significantly with increasing load level, and the creep rate is much greater than that in the secondary creep stage under the same load level.

## 3. Modelling of Creep with Respect to High Load Levels

It was shown by several authors that for low and medium, high load levels, the course of the creep processes up to the secondary creep stage can be well approximated using the following four-element viscoelastic model [19,20], namely

(2) $$\varepsilon =\frac{\sigma_{0}}{E_{1}}+\frac{\sigma_{0}}{E_{2}}(1-e^{-tE_{2}/\eta_{1}})+\frac{\sigma_{0}}{\eta_{2}}t$$

where *ε* is the creep strain, *σ*_0_ is the constant creep stress, *E*_1_ is the elastic modulus, and *E*_2_, *η*_1_, and *η*_2_ are the material parameters. The first term is related to instantaneous elongation at time *t* = 0. With an increase in time, Equation (2) describes an unlimited increase in creep strain, and for *t*→∞, the creep rate is *σ*_0_/*η*_2_. The calibration carried out showed that the values of *E*_2_, *η*_1_, and *η*_2_ strongly depend on the load level and the thickness of the geomembrane. The values of the parameter obtained from the calibration to the individual creep curves show a clear trend. In particular, *E*_2_ increases, and *η*_1_ and *η*_2_ decrease with an increase in load level and the thickness of the geomembrane. This observation gives reason to introduce an appropriate fitting function for each material parameter. It was found that for low load levels up to 40%, a quadratic fitting function, and for load levels 40% < *L* ≤ 60%, a cubic function can well capture the particular load ranges. The fitting parameters for different load ranges and different membrane thicknesses are summarized in Table 4. Here, *L* denotes load level.

(3) $$E_{2}=\left\{\begin{array}{l} 24750L^{2}-17585L+3502.50\;\;L\leq 0.4\\ 13440L^{3}-24944L^{2}+15338.4L-2949.19\;\;L>0.4 \end{array}\right.$$

(4) $$\eta_{1}=\left\{\begin{array}{l} 8750L^{2}-11525L+4037.50\;\;\;\;\;L\leq 0.4\\ -13250L^{3}+27455L^{2}-19556L+5068.68\;\;\;\;\;\;L>0.4 \end{array}\right.$$

(5) $$\eta_{2}=-550177.72L+341070.048\;\;\;\;\;L\leq 0.6$$

(6) $$E_{2}=\left\{\begin{array}{l} 3267L^{2}-2565.5L+664.02\;\;\;\;\;\;L\leq 0.4\\ -1289L^{3}+6010.9L^{2}-5709.2L+1610.04\;\;\;\;\;\;\;\;\;\;\;\;\;\;\;\;\;\;\;\;\;L>0.4 \end{array}\right.$$

(7) $$\eta_{1}=\left\{\begin{array}{l} 750L^{2}-1085L+797.50\;\;\;\;\;\;\;\;\;\;\;\;\;L\leq 0.4\\ -1584L^{3}+4580L^{2}-4875.1L+1862.10\;\;\;\;\;L>0.4 \end{array}\right.$$

(8) $$\eta_{2}=-235799.12L+156521.089L\leq 0.6$$

**Figure 13:**
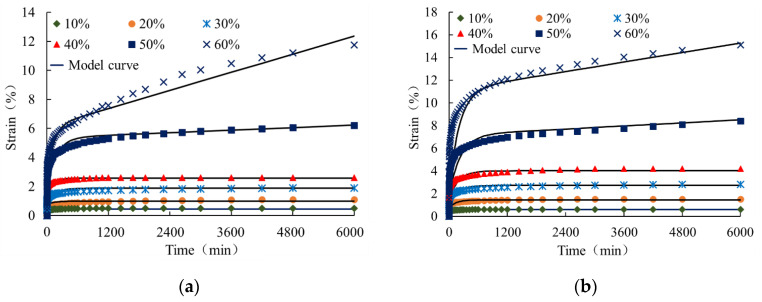
Creep curves under low and medium, high load levels for element thickness of (**a**) 0.5 mm and (**b**) 1.5 mm. (Shapes are experimental data, and solid curves are obtained from the four-element viscoelastic model).

Figure 13 shows the fitting of creep curves under different load levels using the four-element viscoelastic model (2) and the material parameters of Table 4.

It is obvious that the mathematical model (2) can capture rather well the instantaneous, primary, and secondary creep stages. For higher load levels, however, the description of the tertiary creep stage up to the breaking state requires an extension of the four-element model (2). To this end, a term similar to the one proposed by Segard et al. [27] is added. The original term has the following structure:

(9) $$\varepsilon =a\frac{t_{N}^{b}}{1-t_{N}^{c}}$$

where *a*, *b*, and *c* are the material parameters. The standardized time is defined as the ratio of creep time to the time when creep fracture occurs with a range 0 < *t*_N_ < 1. The curve described by Equation (9) is flat when *t*_N_ is small but rises rapidly when *t*_N_ tends to 1. With respect to the experimental data from the present research, it was found that a better adaptation of the creep curve can be obtained when relation (9) is proportional to stress level *σ*_0_ and when standardized time *t*_N_ is replaced by

(10) $$t_{s}=\frac{t}{1.15t_{cr}}$$

Here, *t*_s_ is a dimensionless quantity depending on the current time (*t*) and critical creep time (*t*_r_). Factor 1.15 in the denominator of relation (10) is chosen a little higher than the maximum failure time of the geomembrane, which is approximately 1.07~1.13 times critical creep time (*t_c_*_r_), as shown in Table 2. The improved expression for the additional term is then as follows:

(11) $$\varepsilon =\frac{\sigma_{0}}{\psi }\frac{t_{s}^{n}}{1-t_{s}^{m}}$$

where *ε* is creep strain, *Ψ*, *m*, and *n* are material parameters. By adding the revised relation (11) to the classical four-element model (2), the following five-element viscoelastic model relevant for high load levels is obtained:

(12) $$\varepsilon =\frac{\sigma_{0}}{E_{1}}+\frac{\sigma_{0}}{E_{2}}(1-e^{-tE_{2}/\eta_{1}})+\frac{\sigma_{0}}{\eta_{2}}t+\frac{\sigma_{0}}{\psi }\frac{t_{s}^{n}}{1-t_{s}^{m}}$$

**Figure 14:**
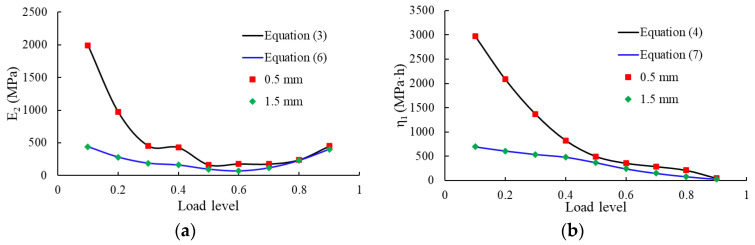

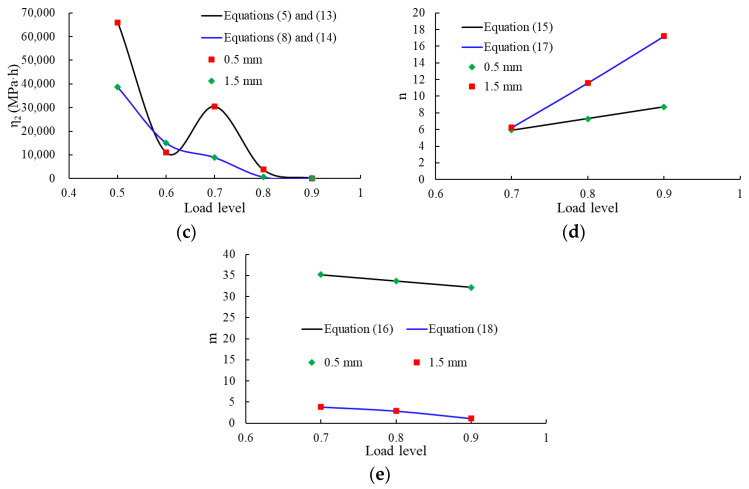
Course of parameters depending on the load level for membrane thicknesses of 0.5 mm and 1.5 mm, respectively (shapes denote experimental data, solid curves denote the fitting data): (**a**) E_2_; (**b**) η_1_; (**c**) η_2_; (**d**) n; (**e**) m.

The corresponding material parameters can be obtained by appropriate approximation functions in a similar manner as shown for the low, medium, and high load levels. In particular, the fitting of the approximation functions is based on the three experiments carried out under the load levels of 70%, 80%, and 90% of the maximum stress peak. For geomembrane thicknesses of 0.5 mm and 1.5 mm and for load levels of 70% ≤ *L* ≤ 90%, the corresponding material parameters are summarized in Table 5. Figure 14 shows the course of the parameters depending on the load level.

(13) $$\eta_{2}=1151610L^{2}-1994064L+862026.54L\geq 0.7$$

(14) $$\eta_{2}=380690L^{2}-653570L+279866L\geq 0.7$$

(15) n=13.9L−3.7967

(16) m=−15.1L+45.77

(17) $$n=\left\{\begin{array}{l} 53.6L-31.27\;\;\;\;L=0.7,0.8\\ 53.6L-31.02\;\;\;\;L=0.9 \end{array}\right.$$

(18) $$m=\left\{\begin{array}{l} -9.5L+10.50L=0.7,0.8\\ -9.5L+9.67L=0.9 \end{array}\right.$$

**Figure 15:**
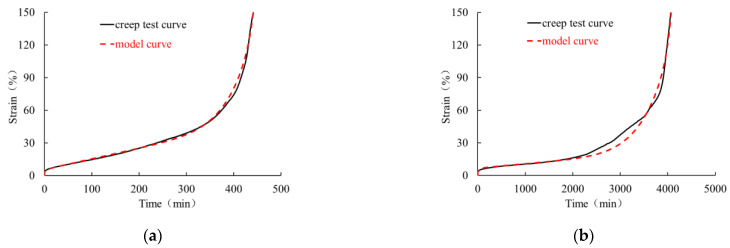

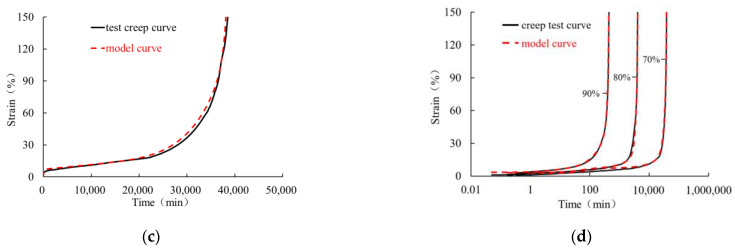
Comparison of creep tests (solid curves) and simulations (dashed curves) of a 0.5 mm thick HDPE geomembrane under a load level of (**a**) 90%, (**b**) 80%, (**c**) 70%, and (**d**) representation in a semilogarithmic coordinate system.

**Figure 16:**
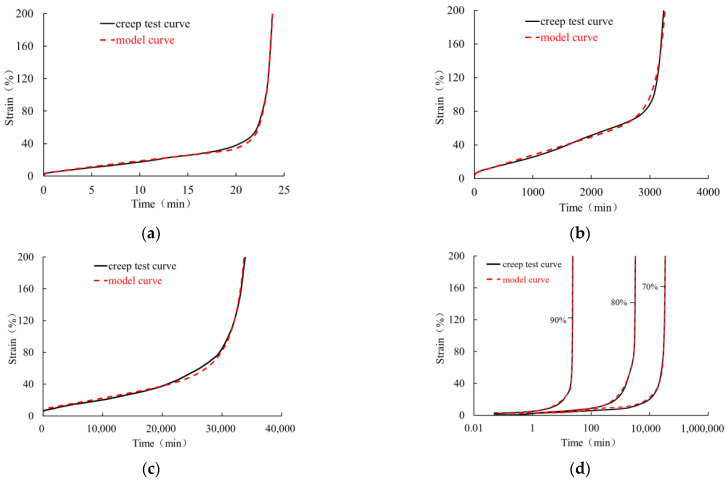
Comparison of creep tests (solid curves) and simulations (dashed curves) of a 1.5 mm thick HDPE geomembrane and a load level of (**a**) 90%, (**b**) 80%, and (**c**) 70%; (**d**) representation in a semilogarithmic coordinate system.

Figure 15 and Figure 16 show the comparison between the experimental data and the curves obtained with the numerical model. The simulations with the extended model (12) are in good agreement with the experimental creep curves, indicating that the improved mathematical model can reasonably reflect the whole creep behavior of the geomembrane under high load levels. In the logarithmic time scale, the model curves and test curves under different high load levels display the characteristics of the initial period of flatness and the rapid rise in strain in the tertiary stage up to creep rupture. Thus, for high load levels, the improved mathematical description (12) can capture the initially slow creep process as well as the non-linear rapid increase in creep strain after passing the secondary stage.

## 4. Conclusions

In this study, the creep behavior of HDPE geomembrane specimens was investigated in tension tests and creep tests under different constant load levels. Particular attention was paid to creep failure behavior under high load levels, namely under 70%, 80%, and 90% of maximum peak stress. To investigate the effects of size on the mechanical response, experiments with two different membrane thicknesses were conducted. A refined mathematical model was proposed to simulate the whole process of different creep characteristics under low, medium, and high load levels. With respect to the assumption of nominal stresses and engineering strains, the following main conclusions can be drawn:

1. Displacement-controlled tensile tests under constant elongation velocity show that after the stress peak, the material undergoes strain softening and subsequently strain hardening up to the breaking state.
2. For different membrane thicknesses, the stress–strain curves are slightly different. Such a size effect can be explained by the inhomogeneous evolution of the microstructure of the material, particularly when the local necking of the membrane specimen becomes dominant.
3. The creep tests carried out show that the creep characteristic is strongly dependent on the applied load level. Under high load levels, the geomembrane experienced the tertiary creep stage, which did not occur under low and medium load levels. From the Sherby–Dorn plot, it can be concluded that the creep rate reaches the minimum value in the secondary creep stage and increases rapidly in the tertiary creep stage. The creep rate of the rapid creep growth stage is much greater than that in the secondary creep stage. The value of creep strain in the tertiary creep stage accounted for more than 80% of strain when creep rupture occurs. For higher load levels, the so-called critical creep time related to a bilinear approximation is lower. It was found that for very high load levels, the amount of the critical creep time and failure time does not necessarily follow the expended trend. Therefore, in creep tests, significant size effects can also be detected under higher load levels.
4. For low, medium, and high load levels, refined fitting functions are proposed, which permit the simulation of the individual creep characteristics within the whole range of particular load levels.

## Figures and Tables

**Table 1:** Properties of HDPE geomembranes.

Thickness (mm)	Density (g/cm^3^)	Peak Strength (MPa)	Peak Elongation (%)	Breaking Strength (MPa)	Breaking Elongation (%)
0.5	0.94	11.42	12.49	10.08	506.38
1.5		10.50	17.00	10.45	610.32

**Table 2:** Characteristic creep values under high load levels for 0.5 mm thick and 1.5 mm thick HDPE geomembranes.

Thickness	Load Level (%)	Critical Strain (%)	Strain at Break (%)	Critical Time (min)	Failure Time (min)	*t_f_*/*t_cr_*
0.5 mm	90	43.40	193.1	404.68	453.22	1.12
	80	27.11	195.7	3694.39	4063.33	1.10
	70	20.92	187.7	34,244.75	38,849.50	1.13
1.5 mm	90	39.57	277.5	22.53	24.02	1.07
	80	54.76	280.7	3075.96	3312.50	1.08
	70	46.08	274.2	31,352.28	34,630.00	1.10

**Table 3:** Creep velocity in stages B–C and stages D–E for 0.5 mm thick and 1.5 mm thick HDPE geomembranes.

Thickness	Load Level (%)	Creep Velocity in Stages B–C (mm/h)	Creep Velocity in Stages D–E (mm/h)
0.5 mm	90	6.016	150.053
	80	0.334	22.688
	70	0.034	1.569
1.5 mm	90	80.07	9018.02
	80	1.020	54.727
	70	0.079	3.716

**Table 4:** Creep parameters of geomembranes under load levels L in the range of 10% ≤ *L* ≤ 60%.

Thickness	Load Level (%)	*E*_1_ (MPa)	*E*_2_ (MPa)	*η*_1_ (MPa·h)	*η*_2_ (MPa·h)
0.5 mm	10	301.46	Equation (3)	Equation (4)	/
	20				/
	30				/
	40				/
	50				Equation (5)
	60				
1.5 mm	10	301.46	Equation (6)	Equation (7)	/
	20				/
	30				/
	40				/
	50				Equation (8)
	60				

**Table 5:** Creep parameters of geomembranes under high load levels of 70% < *L* ≤ 90%.

Thickness	Load Level (%)	*E*_1_ (MPa)	*E*_2_ (MPa)	*η*_1_ (MPa·h)	*η*_2_ (MPa·h)	*Ψ*	*n*	*m*
0.5 mm	90	301.46	Equation (3)	Equation (4)	Equation (13)	7.76	Equation (15)	Equation (16)
	80							
	70							
1.5 mm	90	301.46	Equation (6)	Equation (7)	Equation (14)	14.52	Equation (17)	Equation (18)
	80							
	70							

## Data Availability

Data are contained within the article.
